# Development and validation of a questionnaire assessing volitional competencies to enhance the performance of physical activities in chronic low back pain patients

**DOI:** 10.1186/1471-2474-12-111

**Published:** 2011-05-25

**Authors:** Céline Mathy, Jean-Paul Broonen, Yves Henrotin, Marc Marty, Valérie Legout, Stéphane Genevay, Bernard Duplan, Thierry Bazin, Françoise Laroche, Bernard Savarieau, Christine Cedraschi

**Affiliations:** 1 Social Psychology Unit, Université Libre de Bruxelles, Brussels, Belgium; 2 Belgian Back Society, rue St Luc, Bouge, Belgium; 3 Unit of University Guidance, University of Liege, Liege, Belgium; 4 Bone and Cartilage Research Unit, University of Liege, Institute of Pathology, CHU Sart Tilman, Liege, Belgium; 5 Hôpital Henri Mondor, Créteil, France; 6 Laboratoires Grunenthal, Paris, France; 7 Division of Rheumatology, University Hospitals of Geneva, Hôpital Beau-Séjour, 26, avenue Beau-Séjour, 1211 Genève 14, Switzerland; 8 Hôpital Reine Hortense, Centre hospitalier d'Aix-les-bains, Aix-les-bains, France; 9 Centre Médical Dupic, Lyon, France; 10 Centre d'évaluation et de traitement de la douleur, Hôpital Saint-Antoine, Paris, France; 11 NUKLEUS, Paris, France; 12 Multidisciplinary Pain Center, Division of Clinical Pharmacology and Toxicology & Division of General Medical Rehabilitation, Geneva University Hospitals, Geneva, Switzerland

## Abstract

**Background:**

Motivation has long been emphasized as the most important determinant of action. However, there is a substantial gap between people's goals and their attainment. Patients may be motivated and yet unable to take action if their volitional competencies are insufficient. One of the important tasks of volition is goal-maintenance. Research has stressed the importance of a volitional tool, the implementation intentions. Implementation intentions indicate where, when, and how the action leading to the goal will be performed. Forming implementation intentions favours the execution of goal-directed efforts, and reinforces the relationship between intentions and behaviours. Results from various studies clearly suggest that volitional competencies and implementation intentions could play a role in low back pain (LBP) patients. However, there is at present no questionnaire allowing assessing the capacity of implementation intentions of physical activities in LBP patients.

**Methods/Design:**

This study will develop such a questionnaire, using a 3-step approach. A first qualitative step to build categories and generate items; 30 patients suffering chronic LBP will be invited to participate in semi-structured interviews; verbatim and derived items will then be submitted to a panel of experts, using a Delphi method; a second quantitative step to examine the properties of items, and determine the factorial structure of the questionnaire; 100 patients suffering chronic LBP will be recruited to respond to this phase; and third, preliminary psychometric analyses (item-scale correlations, construct validity, reliability); 180 chronic LBP patients will be recruited for this phase of the study. The relationships between implementation intentions and variables affecting physical activity on chronic LBP patients, i.e. pain, physical capacities, fear-avoidance beliefs, kinesiophobia, work status, and level of physical activity will be considered.

**Discussion:**

Developing a questionnaire to assess implementation intentions would allow investigating the role of these intentions in the transition from acute to chronic LBP. The results of this study should contribute to the understanding of the psychological processes at stake in the development of chronic LBP, and in particular to the identification of factors eventually favouring patients' participation in and adherence to active physical treatments.

## Background

Advice to stay active and continue daily activities, if possible including work, is strongly recommended in patients suffering low back pain (LBP) to avoid chronicity and to improve both the functional and socio-professional status of chronic LBP [1]. In this context, it is important to highlight a biopsychosocial multidisciplinary approach favouring the resumption of physical activities along with patient information and reassurance. However, it may be an issue to obtain LBP patients' participation in an active treatment. Thus, it is important to identify factors favouring participation and adherence to such treatments.

Motivation has long been emphasized as the only or most important determinant of action initiation [2]. Goal theories underline intention as the motivational key determinant of behaviour [3]. For instance, in the theory of planned behaviour [4], intention to perform a behaviour is the decision to act in a particular way and an indication "of how hard people are willing to try, or how much of an effort they are planning to exert, in order to perform the behaviour". Intention is usually measured by endorsement of items such as 'I have the intention to do X'. Intention mediates the influence of three predictors of intentions, namely attitude towards the behaviour, subjective norm and perceived behavioural control (PBC). Attitude toward the behaviour refers to the person's evaluation (favourable vs unfavourable) of the behaviour (e.g., 'Exercise is good for recovering from pain'). Subjective norm refers to the perceived social pressure to perform the behaviour (e.g. 'My wife thinks I ought to exercise'). Finally, perceived behavioural control captures the individual's confidence that he/she is capable of performing the behaviour under investigation (e.g., 'It's up to me to exercise'). These motivational variables culminate in the formation of behavioural intentions (e.g., 'I intend to exercise twice a week').

Meta-analyses indicate that attitude, subjective norm, and PBC account for substantial variance in intentions [e.g. [5]]). But the prediction of behaviour is less impressive, with intention explaining about 28% of the variance in goal achievement [6]. In other words, there is a substantial gap between people's goal or intention and their subsequent attainment [7]. In particular, a patient may be very motivated and yet unable to take action if his/her volitional competencies are insufficient [8]. Volitions have been defined as "special mental events or activities by which an agent consciously and actively exercises his agency to voluntarily direct his thoughts and actions" [9]. Volitional efficiency refers to the actual control over behaviour; consequently, a measure of volitional competencies is hypothesized to increase the explained variance of behaviour over the amount of variance due to the strength of beliefs.

One of important tasks of volition is goal-maintenance [10]. In some contexts, the individual has to overcome difficulties of enactment: habits, competing motivations, beliefs, or underarousal may interfere with the performance of goal-directed action. To overcome these executive difficulties, the individual has to activate his or her volitional competencies, i.e., "the cognitive and emotional processes that govern actual control over behavioural enactment" [11], such as attention control, motivation control, emotion control, self-determination, planning, action initiating, impulse control or volitional optimism [12].

In the last decade, research has given prominence to another volitional tool, namely the implementation intentions. Formation of implementation intentions directed towards a specific objective has been shown to lead to better goal attainment than the mere definition of objectives. Implementation intentions are cognitive schemes that indicate where, when, and how the action leading to the goal will be performed [13,14].

Goal intention has to be distinguished from implementation intention: while goal intention is the focal point of the pre-decisional phase, implementation intention refers to action and the post-decisional phase [15]. Goal intention has been defined as the final stage the individual wants to reach; as such, it transforms wishes into binding objectives, formulated as 'I want to reach X' (e.g. 'I want to do physical exercise').

Implementation intentions have the format of if-then plans: 'If situation X arises, then I will do Y'. Thus, in the case of the goal intention 'I want to do physical exercise' a supporting if-then plan could be 'If one of my friends suggests playing tennis tomorrow, then I will accept' [16]. Implementation intentions create a mental link between a selected cue or situation (e.g. meeting X on Mondays and Fridays) and a goal-directed response (e.g. go to the gym with X). The mental link created by an implementation intention is expected to facilitate goal attainment insofar as forming an implementation intention commits the individual to perform this goal-directed response as soon as the specified situation is encountered. Forming implementation intentions not only favours the execution of goal-directed efforts, but it also allow to protect these efforts and thus to help goal attainment. Indeed, some internal states, such as desires and fears, are known to jeopardize goal attainment. Patients can multiply implementation intentions aiming to decrease the impact of negative internal states in order to facilitate goal striving and increase goal attainment [14,16].

Furthermore, forming implementation intentions reinforces the relationship between intentions and behaviours; it may thus lead to increase participation in physical activities in the general population [17-19]. A recent study showed that interventions incorporating implementation intentions and text message reminders directed at one's walking-related plans or goals increased walking, without reducing other physical activity [20]. These implementation intentions are particularly efficient when the goal is difficult to reach or implies an unpleasant task [14,16]. The efficiency of inducing implementation intentions has been demonstrated in the resumption of physical activities in patients suffering spinal cord injuries [21] or myocardial infarction [22].

These results suggest that implementation intentions could play a role in LBP patients in order to break the vicious cycle leading to chronic LBP by promoting a return to sustained physical activity. The development of implementation intentions may be effective in combating kinesiophobia. To the best of our knowledge, this question has not yet been examined. Indeed, some authors examined intention to exercise in back pain sufferers [23,24] but implementation intentions were not taken into account within the model they used. Additionally, the results of their study are inconsistent, possibly due to the sample size (which had a different power to detect associations among the variables) or because patients with low back pain were not particularly chronic and were not analyzed separately.

There is at present no tool allowing for the assessment of implementation intentions of physical activities in chronic LBP patients. However, such a tool is necessary to appraise possible volitional competencies and to follow-up the results of therapeutic programs aimed to develop such competencies.

## Methods/Design

### Aims

Our main objective is to assess the impact of implementation intention interventions on adherence to exercise programs among patients with chronic low back pain. To do so, this study aims to develop and to validate a questionnaire for measuring the various dimensions of volition. This new instrument will allow the investigation of the capacity of implementation intentions of physical activities of patients with LBP. The relationships between implementation intentions and variables affecting physical activity on chronic LBP patients, i.e. pain, physical capacities, fear-avoidance beliefs, kinesiophobia, work status, and level of physical activity will be considered.

**Figure 1:**
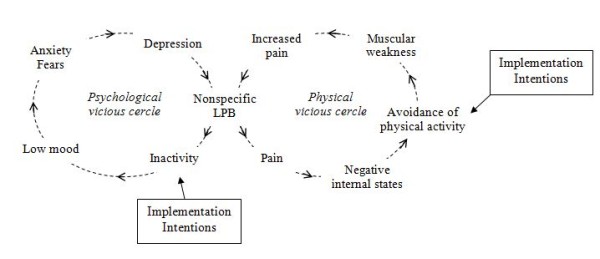
**Psychological and physical vicious circles leading to inactivity (adapted from Arthritis Research Campaign, 2007 **[45]**)**

Furthermore, fear of pain and erroneous beliefs about physical activity have been described as negative internal states which may lead the patient to avoid physical activity and thus to reduce adherence to treatment. Developing a questionnaire to assess implementation intentions would allow investigating the role of these intentions in the transition from acute to chronic LBP (Figure 1).

### Hypotheses

**Figure 2:**
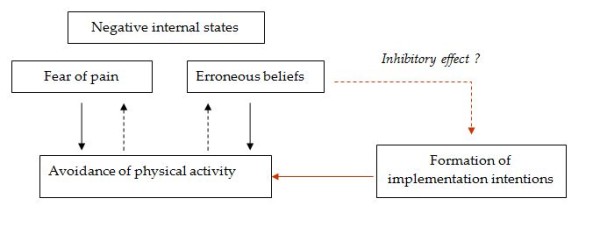
**Hypothetical relationships between the study variables**.

Several hypotheses justify this work. We expect that (a) patients having developed volitional competencies would report more physical activities and that (b) patients having formed implementation intentions would report more physical activities than those who have not formed such intentions (Figures 1 and 2).

We also suppose that (c) resumption of physical activity linked to the actual control of exercises by volitional competencies, especially by implementation intentions, would have a positive effect on negative internal states so that fear of pain and erroneous beliefs about physical activity decrease or even disappear (Figure 2).

We finally hypothesize that (d) erroneous beliefs about physical activity have an inhibitory effect on the formation of volitional competencies, particularly on implementation intentions (Figure 2); and that (e) the positive effect of resuming physical activity would decrease anxiety and depression in the patients (Figure 1).

### Design

The questionnaire will be developed using a 3-step approach [25,26]. A first qualitative step to allow defining the constructs of volitional competencies, in particular the implementation intentions, and the contents of the questionnaire, elicit verbatim remarks, build categories, and generate items; second, a quantitative step to examine the properties of items, and determine the dimensional and factorial structure of the questionnaire; and third, preliminary psychometric analysis (item-scale correlations, construct validity, reliability).

#### 1) Qualitative step

##### 1.a. Defining the concept

Psychologists, rheumatologists, specialists of functional rehabilitation, and patients suffering chronic LBP contribute to reframe the constructs of volitional competencies and implementation intention within the context of chronic LBP and physical activities. The constructs of volitional competencies and implementation intention used to develop the questionnaire will be supported by a systematic review of the literature on this topic. The aspects to be explored will be drawn from the data available in the main electronic databases (in particular the Cochrane Library, Medline, Embase and PsychInfo) between 1999 and 2010. Semi-structured interviews will be constructed at the end of this stage in order to proceed to the next step.

##### 1.b. Generating items

*Participant samples*: thirty patients suffering LBP for more than 12 weeks and consulting rheumatologists, specialists of functional rehabilitation, or orthopaedic surgeons, chosen for their ability to elicit comprehensive and relevant items, will be invited to participate in semi-structured interviews. A multidisciplinary group of eight health professionals including psychologists, rheumatologists and specialists of functional rehabilitation, regularly involved in the treatment of chronic LBP patients, will also respond to semi-structured interviews.

*Data collection*: the semi-structured interviews will be conducted by a psychologist trained in interview procedures. The interviews will be audio-taped and then fully transcribed.

*Content analysis and item generation*: a content analysis will be performed on the verbatim transcripts of the interviews, using a manual data indexing technique to identify key categories. Content analysis will be performed by two psychologists, trained in qualitative procedures and then completed by data from software such as *Alceste *and *Tropes*. This will be followed by a discussion and comparison of the readings of the data, which will be subsequently used to establish analytical categories, as it is the rule in qualitative analyses [27-30]. These categories will then serve as the basis for a final grid, which will be used to analyze the transcripts.

*Item selection*: verbatim and derived items will then be submitted to a panel of experts for selection, using a Delphi method [31]. A group of fifteen French-speaking experts, (Swiss, French and Belgian), members of the Spine Section of the French Society of Rheumatology, will be questioned about item content and relevance. Response modalities, final wording of items and instructions to those completing the questionnaire will also be determined through experts' consensus.

A first version of the questionnaire investigating volition and exercise in back pain patients (VEBPQ1: Volition Exercise Back Pain Questionnaire) will be edited at the end of this qualitative step.

#### 2) Quantitative step 1: determining the factorial structure of the questionnaire

##### Participants

100 patients suffering LBP for more than 12 weeks, with no previous back surgery, free of any other disabling disorder, and French-speaking will be included and recruited according to the type of treatment, i.e. medical consultation alone, active physical therapy, or surgical candidate.

##### Data collection

Patients will be asked to fulfil a demographic questionnaire along with the first version of the VEBPQ at inclusion and two weeks later to allow for reliability analyses.

##### Statistical analyses

The selection and reduction of items will be performed essentially by means of a principal component analysis (PCA). Distribution of responses to the various items will be investigated in order to verify whether all modalities of response have been used and to assess the possible presence of floor and ceiling effects. Dimensionality and factorial structure will also be investigated using an exploratory PCA. The number of factors to be retained will be determined by means of Kaiser criterion (eigenvalue >1) and the screenplot of eigenvalues. Orthogonal rotations (varimax) and then oblique rotations (promax) will then be carried out.

A second version of the questionnaire (VEBPQ2) will be edited at the end of this quantitative step.

#### 3) Quantitative step 2: psychometric analysis of the questionnaire (construct validity and reliability)

##### Patients

180 chronic LBP patients will be recruited for this phase of the study. Inclusion and exclusion criteria as well as recruitment will be similar as in the first two steps.

##### Statistical analyses

Scales' convergent and discriminant validity will be assessed using confirmatory factorial analysis [32,33]. There is no instrument investigating volition within the context of physical activities in chronic LBP patients. Thus, external construct validity cannot be formally assessed. However, correlations will be measured with various dimensions by means of questionnaires or clinical indexes. Pain intensity (Numerical Rating Scale [34]), consequences of pain on daily life (Dallas Pain Questionnaire [35]), use of coping strategies against pain (Coping Strategies Questionnaire [36]), activity-related beliefs and fears (Fear-Avoidance Beliefs Questionnaire [37,38]), kinesiophobia (Tampa Scale for Kinesiophobia [39]), anxiety and depression (Hospital Anxiety and Depression Scale [40,41]), and the Sorensen test to evaluate endurance in extensor muscles of the trunk [42].

##### Data collection

Patients will be asked to fulfil the second version of the VEBQ along with the validated questionnaires allowing to probe into the construct validity of the VEBQ. They will be mailed the VEBQ again two weeks later to allow for reliability analyses. Patients experimenting changes in their clinical status in-between will be excluded from this analysis.

Reliability of the scales will be assessed by composite reliability coefficients [43] and by the Bland and Altman graphical method [44].

This study will be carried out by a multidisciplinary team also involving members of the Belgian Back Society (BBS) and of the "Section Rachis", a working group acknowledged by the French Society of Rheumatology (SFR). This will allow for teamwork within the context of the Back Pain Group of the SFR and for reaching to a large multidisciplinary expert panel.

### Ethics

The study will be conducted in France, Belgium and Switzerland in compliance with the principles of the Helsinki Declaration and the protocol of Good Clinical Practices as well as in accordance with the ethic and regulatory national laws. At each step of the study, patients and physicians will have to give their written consent to participate after being informed about the study protocol. Data collected will be confidential.

This study was approved by the Ethics Committee from the University of Liege and had the Belgian number B707201110271.

## Discussion

In this paper we presented the rationale and the methods for the development of a questionnaire allowing for the appraisal of volitional competencies and of implementations intentions to perform physical activities in chronic LBP patients. This appraisal raises various issues pertaining to different fields, i.e. psychology, rheumatology, and functional rehabilitation. Furthermore, chronic LBP may trigger disabling physical and psychological consequences for the patient; it also generates important economical challenges, both at the personal and the social level. The results of this study should contribute to the understanding of the psychological processes at stake in the development of chronic LBP, and in particular to the identification of factors favouring patients' participation in and adherence to active physical treatments. The development of a questionnaire allowing for the utilization of the constructs of volitional competencies and of implementation intentions in the field of chronic LBP should thus be useful for researchers and therapists as well.

## Competing interests

The authors declare that they have no competing interests.

## Authors' contributions

JPB, CM, YH, CC, MM, SG, BD, TB and FL participated in study concept and design. CC, JPB, CM, YH, MM and VL drafted the manuscript. JPB, YH, CC, CM and MM were involved in critical revision of the manuscript for important intellectual content. YH and VL obtained funding. All authors read and approved the final manuscript.

## Pre-publication history

The pre-publication history for this paper can be accessed here:

http://www.biomedcentral.com/1471-2474/12/111/prepub
